# The association between exposure to psychosocial work factors and mental health in older employees, a 3-year follow-up study

**DOI:** 10.1007/s00420-017-1261-8

**Published:** 2017-09-18

**Authors:** Bo M. Havermans, Cécile R. L. Boot, Trynke Hoekstra, Irene L. D. Houtman, Evelien P. M. Brouwers, Johannes R. Anema, Allard J. van der Beek

**Affiliations:** 1 Department of Public and Occupational Health, EMGO Institute for Health and Care Research, VU University Medical Center, PO Box 7057, 1007 MB Amsterdam, The Netherlands; 2 Body@Work, Research Center Physical Activity, Work and Health, TNO-VU University Medical Center, Amsterdam, The Netherlands; 3 Department of Epidemiology and Biostatistics, EMGO Institute for Health and Care Research, VU University Medical Center, Amsterdam, The Netherlands; 4 Netherlands Organisation for Applied Scientific Research, TNO, Leiden, The Netherlands; 5 School of Social and Behavioural Sciences, Tilburg University, Tranzo, Tilburg, The Netherlands

**Keywords:** Psychosocial, Employee, Mental health, Longitudinal, Exposure

## Abstract

**Purpose:**

Unfavourable exposure to psychosocial work factors threatens older employees’ mental health, and their sustained employment. This study assesses whether an improved compared to stable unfavourable and stable favourable exposure to psychosocial work factors is associated with a change in mental health in older employees at 3-year follow-up.

**Methods:**

The current study used data from the Study on Transitions in Employment, Ability and Motivation (STREAM), in workers aged 45–65 years (*n* = 5249). Two-year (2010–2012) exposure was assessed for psychological demands, autonomy, support, mental load, and distributive justice. Linear regression analyses were performed to compare improved exposure to unfavourable psychosocial work factors with stable unfavourable and stable favourable exposure and mental health at follow-up (2013), corrected for confounders. Analyses were stratified for age groups (45–54 and 55–65 years) and gender.

**Results:**

In certain subgroups, stable unfavourable exposure to psychological demands, autonomy, support, and distributive justice was associated with a significantly lower mental health score than improved exposure. Stable favourable exposure to support was associated with a higher mental health score than improved support, whereas stable favourable exposure to autonomy was associated with a lower mental health score compared to improved exposure.

**Conclusions:**

There is a longitudinal association between changes in exposure to psychosocial work factors and mental health. Improvement in unfavourable exposure to psychosocial work factors was associated with improved mental health. This is important information for organisations that consider deploying measures to improve the psychosocial work environment of older workers.

**Electronic supplementary material:**

The online version of this article (doi:10.1007/s00420-017-1261-8) contains supplementary material, which is available to authorized users.

## Introduction

Psychosocial work factors can be defined as social characteristics of the work environment that interact with individual, psychological factors (Theorell 2007), and are represented in influential work stress models, such as Job Demand-Control (-Support) Model (Johnson and Hall 1988; Karasek 1979), and the Job Demands-Resources model (Demerouti et al. 2001). These psychosocial work factors are associated with work stress, which can result in mental health problems (Netterstrom et al. 2008). Mental health problems in employees account for a substantial part of sickness absence (Henderson et al. 2005). Apart from personal consequences for the employee, this poses a financial burden to both organisations and society at large (Hassard et al. 2014).

Older workers constitute a substantial, and growing part of the total workforce (Ilmarinen 2006). Maintaining a good health status in this occupational group can have a favourable impact on productivity, work ability, and sustainable employment (Leijten et al. 2015). A challenge to maintaining older workers’ good health is exposure to psychosocial work factors (Niedhammer et al. 2013), and a higher prevalence of psychosocial risk factors among older people (Bruce 2002). Shultz and colleagues found that a greater variety of psychosocial factors predicted stress in older workers than in younger workers (Shultz et al. 2010). High psychosocial work stress is associated with elevated depressive symptoms in older workers (Siegrist et al. 2012). Exposure to unfavourable psychosocial work factors is associated with retirement plans and with early retirement in older workers (Thorsen et al. 2012; van den Berg et al. 2010), which puts strain on social security and public welfare (Harper 2014).

The association between unfavourable exposure to psychosocial work factors and mental health outcomes has been assessed in longitudinal organisational stress management evaluation studies, but with relatively short follow-up periods (Westgaard and Winkel 2011). A longer follow-up duration can provide a more comprehensive understanding of the working mechanisms of psychosocial work factors, by allowing for comparison of different exposure patterns that represent changed or stable exposure. Comparing improved exposure to unfavourable exposure can provide insight into potential benefits of actively improving the psychosocial work environment (Boot 2015). Insight into these benefits can motivate employers to try and change detrimental work circumstances, in order to improve the health of their older employees. To assess if improvements made in psychosocial circumstances are sufficient, improved exposure to psychosocial work factors can be compared to stable favourable exposure to psychosocial work factors. If mental health is the same for these two types of exposure, the group with improved exposure resembles the group that reported favourable exposure.

Despite the societal potential, relatively few studies have reported on changes in exposure. Stansfeld and colleagues (Stansfeld et al. 1999) examined the association between improved exposure to psychosocial work factors and risk of psychiatric disorders. They found that, compared to no change, a beneficial change in job demands reduced the risk of psychiatric disorders, and an adverse change increased that risk. Wang and colleagues (Wang et al. 2009) studied improved exposure to psychosocial work factors, and found decreased job strain to be related to a decreased risk of major depression. Finally, de Lange and colleagues (de Lange et al. 2002) found that decreased job strain was associated with higher job satisfaction, but not with improvements in depression and sickness absence. Even though there seems to be a tendency towards improvement of health risks with the decline of unfavourable exposure, findings thus far suggest that more research is needed to enhance our understanding of changed exposure.

In the current study, we assessed the association between improved, stable unfavourable, and stable favourable exposure to psychosocial work factors and changes in mental health in older employees, in a longitudinal research design. The psychosocial work factors studied were psychological demands, autonomy, support, mental load, and distributive justice. The research question of the current study was: is an improved compared to stable unfavourable and stable favourable exposure to psychosocial work factors associated with a change in mental health in older employees after 3 years?

## Methods

### Study design and procedure

The current study used data from the Study on Transitions in Employment, Ability and Motivation (STREAM), an ongoing longitudinal cohort study in the Netherlands, among individuals aged 45–64 years (Ybema et al. 2014). Starting in 2010, participants were approached annually (via email) to fill out online questionnaires, which took approximately 25 min to complete. For every year that participants completed the STREAM questionnaire, they received a small financial incentive (about 3 euros). Initial non-responders were sent a maximum of two reminders. A more detailed description of the STREAM study design has been given elsewhere (Ybema et al. 2014).

#### Participants

**Fig. 1:**
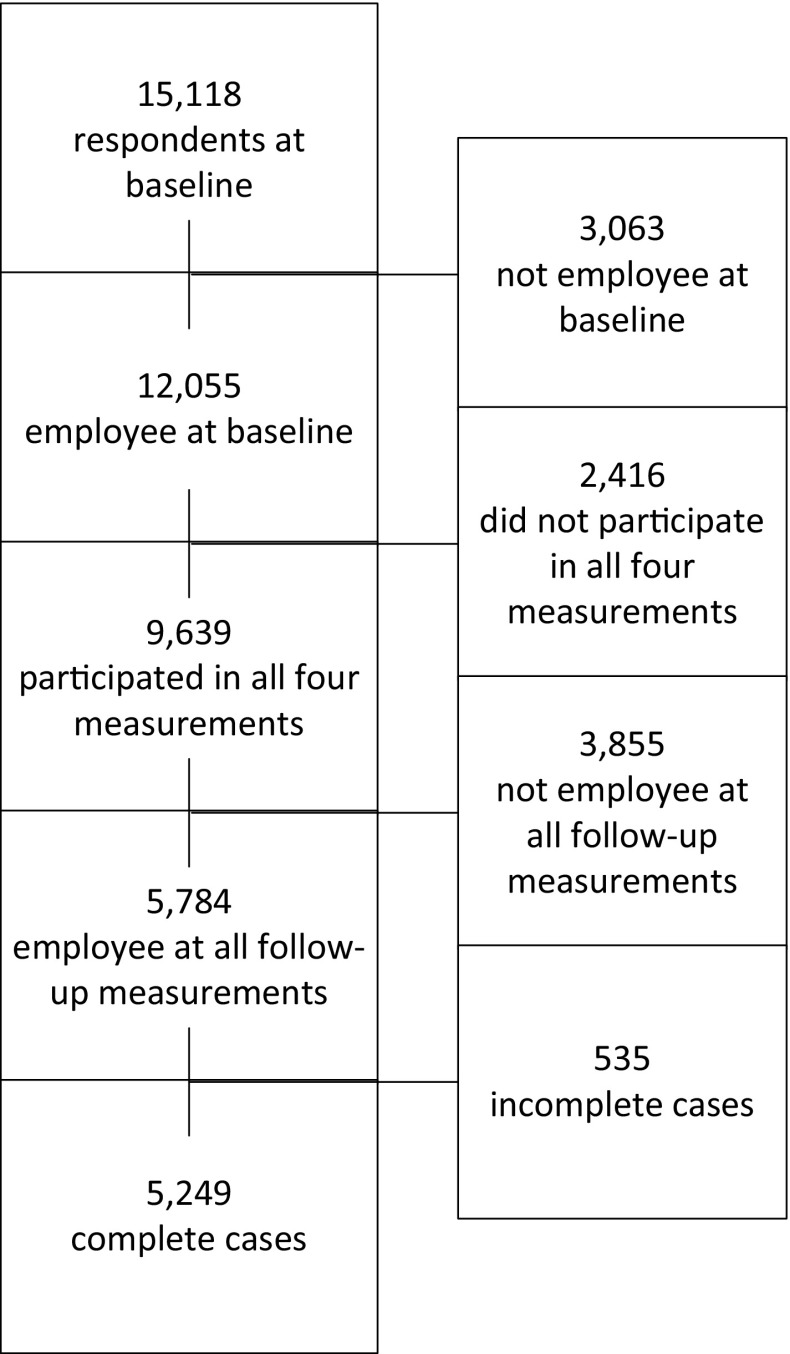
Flow chart of study sample

For the current study, STREAM data were used from the baseline measurement in 2010 (T0) and follow-ups in 2011 (T1), 2012 (T2), and 2013 (T3). At baseline, 15,118 individuals participated (response rate 71%). Of the baseline sample, 82% (*n* = 12,430) responded at T1, 72% (*n* = 10,952) responded at T2, and 64% (*n* = 9,639) responded at T3. The study population consisted of all employees at baseline (*n* = 12,055). They were included in the current study if they had participated in all four measurement moments (*n* = 9639). Participants who were not employee (i.e. unemployed or self-employed) at baseline or any of the follow-ups (*n* = 3765) were excluded, resulting in a sample of 5874. Additionally, persons with missing data on psychosocial work factors at T0–T2 (*n* = 473), and mental health at T3 (*n* = 152) were excluded. The total study population consisted of 5249 participants (see Fig. 1).

### Psychosocial work factors at T0, T1, and T2

Psychological demands were measured using a four-item subscale from the Job Content Questionnaire (JCQ; Cronbach’s alpha at T0 = 0.86) (Karasek et al. 1998) that assesses the individual work experience. Autonomy was measured using a five-item subscale from the JCQ (Cronbach’s alpha at T0 = 0.76) that addresses decision making, ability to determine the order and speed of conducting tasks, coming up with solutions, and being able to take time off. Support is the extent to which colleagues and supervisors are willing to help and listen to work-related problems, and was measured using a four-item subscale of the Copenhagen Psychosocial Questionnaire (COPSOQ; Cronbach’s alpha at T0 = 0.80) (Pejtersen et al. 2010). Mental load was assessed with a three-item subscale from the Netherlands Working Conditions Survey (NWCS; Cronbach’s alpha at T0 = 0.79) (Koppes et al. 2009), assessing the extent to which the job requires much thinking, much attention, and keeping one’s mind on the job. Distributive justice was measured with six items from de Boer et al (2002), about salary and appreciation in relation to the individual’s relative effort, result, and quantity of tasks, compared to colleagues (Cronbach’s alpha at T0 = 0.87). All subscales consisted of items measured on five-point Likert scales (1 “never” to 5 “always” for psychological demands, autonomy, support, and mental load; 1 “much too low” to 5 “much too high” for distributive justice).

### Exposure

For all psychosocial work factors, higher scores were coded to represent more unfavourable exposure, i.e. low autonomy, poor distributive justice, poor support, high psychological demands and high mental load. Due to the lack of meaningful cutoff points for exposure to psychosocial work factors in the existing literature, high risk was defined as the most unfavourable quartile of exposure. Using measurements T0, T1, and T2, participants were divided into three exposure groups: “stable unfavourable”, “improved”, and “stable favourable” (Table 1).

**Table 1:** Exposure groups for psychosocial work factors

Exposure group	Risk at T0^a^	Risk at T1^a^	Risk at T2^a^
Stable unfavourable (*N* = 803)^b^	High	High	High
Improved (*N* = 572)^b^	High	High	Low
	High	Low	Low
Stable favourable (*N* = 3456)^b^	Low	Low	Low

^a^T0 = baseline; T1 = first follow-up; T2 = second follow-up

^b^Maximum group size

### Mental health at T0 and T3

The primary outcome was mental health, as assessed with the Short Form Health Survey (SF-12; Cronbach’s alpha at T0 = 0.84) (Ware et al. 1996). The SF-12 is a twelve-item questionnaire from which a component score was calculated for mental health. The SF-12 contains items such as: “How much time during the past 4 weeks have you felt calm and peaceful? [All of the time (1)—none of the time (6)]” and “During the past 4 weeks, have you, as a result of any emotional problems, accomplished less than you would like? [Yes (1)—No (2)]”. The mental health scale score was standardised using USA 1998 standards, resulting in a possible range from 0 to 100, with a higher score indicating better mental health. Mental health was measured at the last follow-up (T3), corrected for mental health at baseline by adding baseline values of mental health to the model. The SF-12 was found to have an acceptable test–retest reliability and convergent validity (Ware et al. 1995, 1996).

### Analyses

Descriptive analyses were run with baseline data for gender, education, and marital status, and for general health, job security, psychosocial work factors (psychological demands, autonomy, support, mental load, distributive justice), and mental health.

#### Associations

Pearson correlations between the psychosocial work factors (psychological demands, autonomy, support, mental load, distributive justice) and mental health at baseline were calculated. Exposure groups were compared with improved exposure as the reference category. To that end, dummy variables were created for stable unfavourable and stable favourable exposure that allowed comparison of three exposure groups in one analysis. Linear regression analyses were used to assess the association between exposure to the five psychosocial work factors and mental health. First, crude analyses were performed using univariable regression. Then, checks for confounding were performed using multivariable regression. If the association between exposure and mental health changed more than 10% by adding the possible confounder to the crude model, the added variable was included in the model as a confounder. Selection of possible confounders was based on the existing literature (Leijten et al. 2015). Possible confounders were age, gender, education level, marital status (single or not), general health (measured using the SF-12, from which a component score can also be calculated for general health) (Ware et al. 1996), and job security (measured using one item, on a four-point Likert scale; 1 “not present at all” to 4 “highly present” (Ybema et al. 2014). To check for effect modification, interactions of exposure with age and gender were inspected. Effect modification was assumed when interactions were statistically significant (*p* < 0.05) and subgroup analyses were performed. Because the primary outcome, mental health, was not normally distributed, bootstrapped analyses were performed (bias corrected and accelerated; 5000 samples) to check the robustness of the crude and the adjusted models (Efron and Tibshirani 1994).

Loss-to-follow-up analyses were performed for all variables at baseline, comparing employed participants with complete data to those who were employed at T0, but had not participated in at least one follow-up, using Chi square analyses for gender, education, and marital status, and an independent samples *T* test for age, general health, job security, mental health, psychological demands, autonomy, support, mental load, and distributive justice. *p* values were considered significant when they were smaller than 0.05. IBM Statistical Package for the Social Sciences (SPSS, version 20) was used to analyse the data.

## Results

### Study population characteristics

Characteristics of the study population at baseline are presented in Table 2. The sample consisted of more males than females, and the average age was 53.28 years (SD = 4.95). Characteristics of the separate psychosocial work factor exposure groups are presented in the supplementary material (Appendix 1). Drop-out rates were higher among older individuals, and individuals with a low level of education. In Table 3, bivariate correlations between psychosocial work factors and mental health are presented.

**Table 2:** Individual factors, psychosocial work factors, and mental health at baseline

	*n* (%)	Mean (SD)	Median (min–max)^a^
Individual factors			
Gender (female)	2280 (43.4)	–	–
Age	–	53.28 (4.95)	53.00 (45–65)
Education			
Low^b^	1334 (25.4)	–	–
Medium^b^	2059 (39.2)	–	–
High^b^	1856 (35.4)	–	–
Sector/profession			
Craft and industry	333 (6.3)	–	–
Transport	201 (3.8)	–	–
Administrative	841 (16.0)	–	–
Commercial	286 (5.4)	–	–
Services	451 (8.6)	–	–
Health care	773 (14.7)	–	–
Education	475 (9.0)	–	–
Specialist^c^	581 (11.1)	–	–
Agriculture	22 (0.4)	–	–
Executive staff	531 (10.1)	–	–
Other	750 (14.3)	–	–
Marital status: not single	4080 (77.7)	–	–
General health	–	52.11 (7.35)	54.87 (15.33–67.13)
Job security	–	2.91 (0.80)	3.00 (1 low–4 high)
Psychosocial work factors			
Psychological demands	–	3.16 (0.75)	3.25 (1 low–5 high)
Autonomy	–	3.83 (0.69)	4.00 (1 low – 5 high)
Support	–	3.61 (0.75)	3.75 (1 low – 5 high)
Mental load	–	4.21 (0.63)	4.33 (1 low – 5 high)
Distributive justice	–	1.49 (0.50)	1.50 (1 low – 3 high)
Mental health	–	52.58 (7.93)	55.37 (10.13 – 69.43)

*SD* standard deviation

^a^minimum score–maximum score

^b^Low: lower general secondary education, preparatory secondary vocational education, Medium: intermediate vocational training, higher general secondary education, pre-university education, and High: higher vocational education, university education

^c^e.g. statistician, architect, IT specialist, artist

**Table 3:** Correlations between psychosocial work factors and mental health at baseline

	Psychological demands	Autonomy	Support	Mental load	Distributive justice
Mental health	−.11*	−13*	−.02	−.14*	−.11*

*p < 0.01

### Changes in exposure to unfavourable psychosocial work factors and mental health

Crude and adjusted models of the association between exposure to psychosocial work factors and mental health are presented in Table 4. Because of effect modification, stratified results are presented for autonomy (stratified by age), and for support and distributive justice (stratified by gender). In the adjusted models, stable unfavourable exposure was associated with a significantly lower score on mental health compared to improved exposure for psychological demands (*B* = −0.929, CI = −1.698 to −0.159), autonomy (*B* = −1.046, CI = −2.413 to −0.713 in participants aged 45–54 years; *B* = −2.881, CI = −4.090 to −1.672 in participants aged 55–64 years), support in men (*B* = −1.052, CI = −2.092 to −0.012), and distributive justice in women (−1.376, CI = −2.732 to −0.020). Stable favourable exposure was associated with a significantly lower mental health score compared to improved exposure for autonomy in participants aged 55–64 years (*B* = −1.374, CI = −2.427 to −0.321), and with a significantly higher mental health score for support (*B* = 0.865, CI = 0.037–1.693 in men; *B* = 1.075, CI = 0.076–2.074 in women).

**Table 4:** The association between 2-year exposure to psychosocial work factors and mental health at 3-year follow-up, with improved exposure as the reference category

Psychosocial work factors	Exposure	Mental health	
		*B*	95%-CI
Psychological demands (*N* = 4391)			
Crude	Stable favourable	0.808*	0.125 to 1.491
	Stable unfavourable	−1.091*	−1.932 to −0.250
Adjusted^a^	Stable favourable	0.070	−0.557 to 0.696
	Stable unfavourable	−0.929*	−1.698 to −0.159
Autonomy (*N* = 4497)			
Crude	Stable favourable	0.548	−0.179 to 1.275
	Stable unfavourable	−1.563**	−2.413 to −0.713
Adjusted^b^			
Age 45–54^c^	Stable favourable	−0.042	−0.921 to 0.828
	Stable unfavourable	−1.046*	−2.064 to −0.028
Age 55–64	Stable favourable	−1.374*	−2.427 to −0.321
	Stable unfavourable	−2.881**	−4.090 to −1.672
Support (*N* = 4135)			
Crude	Stable favourable	1.674**	0.992 to 2.356
	Stable unfavourable	−0.786	−1.657 to 0.084
Adjusted^d^			
Men^c^	Stable favourable	0.865*	0.037 to 1.693
	Stable unfavourable	−1.052*	−2.092 to −0.012
Women	Stable favourable	1.075*	0.076 to 2.074
	Stable unfavourable	0.386	−0.891 to 1.627
Mental load (*N* = 4399)			
Crude	Stable favourable	0.100	−0.616 to 0.816
	Stable unfavourable	−0.712	−1.665 to 0.241
Adjusted^e^	Stable favourable	−0.186	−0.846 to 0.473
	Stable unfavourable	−0.696	−1.566 to 0.175
Distributive justice (*N* = 4159)			
Crude	Stable favourable	0.813*	0.150 to 1.477
	Stable unfavourable	−0.724	−1.621 to 0.172
Adjusted^f^			
Men^c^	Stable favourable	0.428	−0.357 to 1.212
	Stable unfavourable	−0.078	−1.106 to 0.950
Women	Stable favourable	−0.505	−1.476 to 0.466
	Stable unfavourable	−1.376*	−2.732 to −0.020

*p < 0.05; **p < 0.001

^a^For mental health at baseline

^b^For education level, marital status, general health, job security, and mental health at baseline

^c^Stratification was based on the presence of effect modification

^d^For marital status, age, job security, and mental health at baseline

^e^For gender, education level, marital status, age, general health, job security, and mental health at baseline

^f^For general health, job security, and mental health at baseline

## Discussion

Improved exposure to psychosocial work factors (psychological demands, autonomy, support, and distributive justice) was associated with better mental health compared to stable unfavourable exposure to psychosocial work factors in all older workers. Improved autonomy was associated with better mental health in employees aged 55–64 years, compared to stable unfavourable exposure to autonomy. Improved exposure to support was associated with poorer mental health in men, compared to stable unfavourable exposure.

This study moved past the traditional comparison of workers with a high exposure to those with a low exposure and their respective health outcome risks, by taking into account improved exposure (Boot 2015). Looking at improved exposure has an applied advantage, because it shows characteristics of more specific groups that are relevant in a prevention setting. Building on previous research that has generally shown improvements in health risks with the decline of unfavourable exposure to psychosocial work circumstances (de Lange et al. 2002; Stansfeld et al. 1999; Wang et al. 2009), this study provides specific insight into improved exposure to psychosocial work factors and mental health in older workers. This is important, because older workers constitute a substantial part of the workforce.

### Changes in exposure to psychosocial work factors and mental health

The finding that psychological demands and mental health are associated is corroborated by several studies, linking high psychological demands at work to a higher risk of mental health problems, such as depression and anxiety (Plaisier et al. 2007; Virtanen et al. 2007). High psychological demands were associated with poorer mental health compared to improved demands, which suggests that improvements in psychological demands may be beneficial to older employees’ mental health. There was no difference in mental health between participants with improved exposure to psychological demands and participants with stable favourable exposure.

Stable unfavourable exposure to autonomy was associated with poorer mental health compared to improved exposure. This is not surprising, because earlier studies have reported a positive association between autonomy and mental health (Leijten et al. 2015; Thompson and Prottas 2006). The effect was stronger in employees aged 55–64 years, than for employees aged 45–54 years. This could be explained by findings suggesting that psychosocial risks may be more prevalent in older people (Bruce 2002), and that a greater variety of psychosocial work factors may predict mental health problems in older employees than in younger employees (Shultz et al. 2010). In older employees, there might be more room for improvement. In employees aged 55–64 years, stable favourable exposure autonomy was associated with poorer mental health compared to improved exposure. This finding does not seem to fit numerous other studies, in which autonomy is positively associated with mental health (Bond and Flaxman 2006; Thompson and Prottas 2006). Possibly, in this select group, relatively high autonomy is accompanied by high responsibility, due to seniority. Responsibility can be regarded as a job demand that is associated with mental strain (Karasek 1979).

Stable favourable exposure to support was associated with better mental health compared to improved exposure. This has been reported in earlier studies that showed a positive association between support and mental health in older workers (Leijten et al. 2015), and demonstrated that support protected against the incidence of mental health problems, such as depression and anxiety (Plaisier et al. 2007). In men, stable unfavourable exposure to support showed poorer mental health compared to improved exposure. No difference in mental health was found between improved and stable unfavourable exposure to support in women. Gender differences in effects of social support on mental health have been demonstrated before (Plaisier et al. 2007). Vermeulen and Mustard (2000) suggested that psychosocial work exposures, including social support, may determine psychological well-being more in men compared to women. This is in line with our findings.

Stable unfavourable and stable favourable exposure to mental load showed no differences in mental health compared to improved exposure. This could be explained by a selection effect. Mental load might not be a significant problem for this study sample, due to the ‘healthy worker effect’ (Li and Sung 1999), which is characterised by relatively higher employment among physically and mentally healthier workers, due to competition and selection during hiring procedures, and relatively higher natural outflow out of employment of workers with suboptimal health. As this study focused on older workers, this selection process may have taken place, leaving the mentally more capable (of dealing with the mental load of their work) employed.

In women, stable unfavourable exposure to distributive justice was associated with poorer mental health compared to improved exposure. This is in line with earlier studies, in which distributive justice was negatively associated with depressive symptoms (Ybema and Van den Bos 2010), and burnout (Liljegren and Ekberg 2009). The gender difference in the association between unfavourable exposure to distributive justice and mental health can be explained by the notion that men and women define distributive justice in a different way, with women putting more emphasis than men on the extent to which they were treated with respect and dignity, and the favourability of their outcomes (Kulik et al. 1996). Female employees are structurally treated unfairly when it comes to, for instance, the distribution of a central resource: salary (Arulampalam et al. 2007). The gender pay gap (i.e. a structural imbalance in the distribution of pay, favouring men) illustrates a distributive injustice between men and women. With these structural differences in distributive circumstances, it is plausible that the meaning of distributive justice differs for men and women, as well as its association with mental health.

### Strengths and limitations

A strength of this study is the fact that it used a follow-up period of 3 years. Also, by comparing an improvement in exposure to stable unfavourable and stable favourable exposure, this study gives insight into potential benefits of reducing exposure (Boot 2015), which are relevant in the applied setting of mental health improvement of older employees. Previous studies on changes in psychosocial work factors have mainly focused on job strain, and job control (de Lange et al. 2002; Wang et al. 2009). In this study, a variety of psychosocial work factors was considered, making it possible to discover similarities and differences in the association between different psychosocial work factors and mental health. Even though unfavourable exposure to psychosocial work factors was associated with poorer mental health in many instances, compared to improved exposure, the effects found were rather small. Even though a component mental health score of the SF-12 was used, it might have provided a more general health score than instruments that were developed for specific mental health issues. Possibly, a more specific measurement of mental health issues (such as stress) would have resulted in stronger associations (Singh-Manoux et al. 2006).

A healthy worker effect and common method variance due to the use of self-report measures could not be ruled out in the current study (Li and Sung 1999; Podsakoff et al. 2003). Consequently, the effects of unfavourable exposure to psychosocial work factors may have been masked as our population could represent a relatively healthy selection of older workers. A risk of selection bias exists, because only complete cases were used for analysis, which is most appropriate when values are missing completely at random. As respondents that were excluded due to missing values were slightly older and more likely to be female, missing values did not occur completely at random. This means that there was a potential bias due to the loss of information and precision (Little and Rubin 2014). Stepwise regression was used to check and correct for confounding, with potential confounders being selected based on the existing literature. The decision to include confounders was based on a > 10% change in the regression coefficient. Acyclic directed graphs could have been used to select confounders in a less arbitrary way, based on a theoretical model (Thulasiraman and Swamy 2011). A problem with stepwise regression is that it falsely yields confidence intervals that are too narrow, introducing bias characterised by overestimation of effects (Altman and Andersen 1989; Tibshirani 1996). Another limitation of this study was the absence of meaningful cutoff values for psychosocial work factor exposure. For the sake of readability, we chose the most unfavourable quartile for the qualification of ‘high-risk’ exposure. As a consequence, it is uncertain if those in the high-risk group were actually at high risk. More pronounced effects could be found if the cutoffs were higher in reality. On the other hand, if the cutoffs were lower in reality, associations might have been smaller. In the current study, associations between stable favourable and improved exposure were non-significant for psychological demands, autonomy (in employees aged 45–54), mental load, and distributive justice. This leaves questions unanswered about what magnitude of improvement is needed to get exposure down to levels that are not potentially harmful to older employees. In the future, more meaningful cutoff values should be established. Alternatively, latent trajectories could be distinguished to create change groups, as done by Haukka and colleagues (Haukka et al. 2011). Finally, no causal inferences could be made, because this study only looked at longitudinal associations. Especially because the effect sizes found were small, future studies should confirm whether there is a causal connection between improved exposure to psychosocial work factors, and improvements in mental health in older workers.

### Implications of findings

Cautious interpretation of the findings of this study is advised. Even though many associations were statistically significant, the effects did not approach the Minimal Clinically Important Difference (MCID) value for the mental health component score of the SF-12, which is 4.7 (Parker et al. 2013). Moreover, more definitive answers should be pursued in future research that focuses on causal connections between exposure to psychosocial work factors and changes in mental health in older workers.

Reviews show that organisational-level interventions that target psychosocial work factors can be beneficial to employees’ mental health (van der Klink et al. 2001), but that implementation of these interventions is met with many challenges that could influence findings of intervention evaluations (Egan et al. 2009; Havermans et al. 2016; Montano et al. 2014). The current study shows the dynamics of psychosocial work factor exposure and mental health outside the context of a specific intervention. It shows that if interventions are effective in improving unfavourable psychosocial work factors, this might be associated with an improvement in mental health in older workers.

This could encourage organisations to deploy policies and interventions to improve the mental health of their older employees. For example, by redesigning jobs in such a way, that they fit the psychosocial needs of older employees, work ability could be promoted, and retirement could be postponed. When dealing with prevention of exposure to unfavourable psychosocial work factors, economic realities (such as the budget made available for prevention) should be taken into account. In this regard, practice can be more challenging than theory. The longitudinal perspective taken in this study is relevant for policy makers, because it can give insight into improvements made by improving unfavourable exposure to psychosocial work factors, and into the efforts still required to bring older employees from improved exposure to a level that is comparable to stable favourable exposure. Policy makers should take this into account when planning and funding research and other projects aimed at improving employees’ mental health.

## Conclusion

Improved exposure to psychosocial work factors was associated with better mental health in older employees compared to stable unfavourable exposure. The overall findings indicate that reducing unfavourable exposure to psychosocial work factors may be beneficial to older employees’ mental health. This is important information for organisations that consider deploying measures to manage unfavourable exposure to psychosocial work factors in older workers.

## Electronic supplementary material

Below is the link to the electronic supplementary material.

Supplementary material 1 (DOCX 15 kb)
