# Supplementary material for: The association between exposure to psychosocial work factors and mental health in older employees, a 3-year follow-up study
Source: Int Arch Occup Environ Health. 2017 Sep 18;91(1):57–66. doi: 10.1007/s00420-017-1261-8 (PMC5752729; doi:10.1007/s00420-017-1261-8)
Supplement: Supplementary file 1 — Supplementary material 1 (DOCX 15 kb) [file 420_2017_1261_MOESM1_ESM.docx]

APPENDIX 1:

*Table 1. Group sizes and demographic information for psychosocial work factor exposure groups*

| Psychosocial work factors | | N | Gender (female) | Age | Education^b^ |  |
| --- | --- | --- | --- | --- | --- | --- |
|  | |  | n(%) | Mean (SD)^a^ |  | n(%) |
| *Psychological demands* | |  |  |  |  |  |
|  | Stable favourable exposure | 3173 | 1327(41.8) | 53.53(5.04) | Low  Medium  High | 902(28.4)  1283(40.4)  988(31.1) |
|  | Improved exposure | 528 | 246(46.6) | 53.11(4.91) | Low  Medium  High | 131(24.8)  216(40.9)  181(34.3) |
|  | Stable unfavourable exposure | 690 | 318(46.1) | 52.82(4.71) | Low  Medium  High | 122(17.7)  247(35.8)  321(46.5) |
| *Autonomy* | |  |  |  |  |  |
|  | Stable favourable exposure | 3249 | 1213(37.3) | 53.27(4.98) | Low  Medium  High | 739(22.7)  1250(38.5)  1260(38.8) |
|  | Improved exposure | 445 | 209(47.0) | 52.94(4.93) | Low  Medium  High | 130(29.2)  194(43.6)  121(27.2) |
|  | Stable unfavourable exposure | 803 | 473(58.9) | 53.48(4.73) | Low  Medium  High | 227(28.3)  306(38.1)  270(33.6) |
| *Support* | |  |  |  |  |  |
|  | Stable favourable exposure | 3032 | 1366(45.1) | 52.90(4.91) | Low  Medium  High | 726(23.9)  1204(39.7)  1102(36.3) |
|  | Improved exposure | 526 | 214(40.7) | 53.60(4.95) | Low  Medium  High | 141(26.8)  205(39.0)  180(34.2) |
|  | Stable unfavourable exposure | 577 | 240(41.6) | 54.28(4.86) | Low  Medium  High | 163(28.2)  227(39.3)  187(32.4) |
| *Mental load* | |  |  |  |  |  |
|  | Stable favourable exposure | 3456 | 1553(44.9) | 53.23(4.97) | Low  Medium  High | 1012(29.3)  1429(41.3)  1015(29.4) |
|  | Improved exposure | 475 | 188(39.6) | 53.13(4.86) | Low  Medium  High | 86(18.1)  177(37.3)  212(44.6) |
|  | Stable unfavourable exposure | 468 | 177(37.8) | 53.53(4.87) | Low  Medium  High | 69(14.7)  150(32.1)  249(53.2) |
| *Distributive justice* | |  |  |  |  |  |
|  | Stable favourable exposure | 3097 | 1430(46.2) | 53.46(5.01) | Low  Medium  High | 772(24.9)  1178(38.0)  1147(37.0) |
|  | Improved exposure | 572 | 219(38.3) | 53.05(4.73) | Low  Medium  High | 144(25.2)  232(40.6)  196(34.3) |
|  | Stable unfavourable exposure | 490 | 174(35.5) | 53.27(4.83) | Low  Medium  High | 123(25.1)  211(43.1)  156(31.8) |

^a^SD = Standard Deviation; ^b^Low: lower general secondary education, preparatory secondary vocational education, Medium: intermediate vocational training, higher general secondary education, pre-university education, and High: higher vocational education, university education
